# IFNγ/IL-10 Co-producing Cells Dominate the CD4 Response to Malaria in Highly Exposed Children

**DOI:** 10.1371/journal.ppat.1003864

**Published:** 2014-01-09

**Authors:** Prasanna Jagannathan, Ijeoma Eccles-James, Katherine Bowen, Felistas Nankya, Ann Auma, Samuel Wamala, Charles Ebusu, Mary K. Muhindo, Emmanuel Arinaitwe, Jessica Briggs, Bryan Greenhouse, Jordan W. Tappero, Moses R. Kamya, Grant Dorsey, Margaret E. Feeney

**Affiliations:** 1 Department of Medicine, San Francisco General Hospital, University of California, San Francisco, San Francisco, California, United States of America; 2 Infectious Diseases Research Collaboration, Kampala, Uganda; 3 Center for Global Health, Centers for Disease Control and Prevention, Atlanta, Georgia, United States of America; 4 Department of Medicine, Makerere University College of Health Sciences, Kampala, Uganda; 5 Department of Pediatrics, University of California, San Francisco, San Francisco, California, United States of America; National Institute for Medical Research, United Kingdom

## Abstract

Although evidence suggests that T cells are critical for immunity to malaria, reliable T cell correlates of exposure to and protection from malaria among children living in endemic areas are lacking. We used multiparameter flow cytometry to perform a detailed functional characterization of malaria-specific T cells in 78 four-year-old children enrolled in a longitudinal cohort study in Tororo, Uganda, a highly malaria-endemic region. More than 1800 episodes of malaria were observed in this cohort, with no cases of severe malaria. We quantified production of IFNγ, TNFα, and IL-10 (alone or in combination) by malaria-specific T cells, and analyzed the relationship of this response to past and future malaria incidence. CD4^+^ T cell responses were measurable in nearly all children, with the majority of children having CD4^+^ T cells producing both IFNγ and IL-10 in response to malaria-infected red blood cells. Frequencies of IFNγ/IL10 co-producing CD4^+^ T cells, which express the Th1 transcription factor *T-bet*, were significantly higher in children with ≥2 prior episodes/year compared to children with <2 episodes/year (*P*<0.001) and inversely correlated with duration since malaria (*Rho* = −0.39, *P*<0.001). Notably, frequencies of IFNγ/IL10 co-producing cells were not associated with protection from future malaria after controlling for prior malaria incidence. In contrast, children with <2 prior episodes/year were significantly more likely to exhibit antigen-specific production of TNFα without IL-10 (*P* = 0.003). While TNFα-producing CD4^+^ T cells were not independently associated with future protection, the absence of cells producing this inflammatory cytokine was associated with the phenotype of asymptomatic infection. Together these data indicate that the functional phenotype of the malaria-specific T cell response is heavily influenced by malaria exposure intensity, with IFNγ/IL10 co-producing CD4^+^ T cells dominating this response among highly exposed children. These CD4^+^ T cells may play important modulatory roles in the development of antimalarial immunity.

## Author Summary

Despite reports of decreasing malaria morbidity across many parts of Africa, the incidence of malaria among children continues to be very high in Uganda, even in the setting of insecticide-treated bednets and artemisinin-based combination therapy. Additional control measures, including a vaccine, are sorely needed in these settings, but progress has been limited by our lack of understanding of immunologic correlates of exposure and protection. T cell responses to malaria are thought to be important for protection in experimental models, but their role in protecting against naturally acquired infection is not clear. In this study, we performed detailed assessments of the malaria-specific T cell response among 4-year-old children living in Tororo, Uganda, an area of high malaria transmission. We found that recent malaria infection induces a malaria-specific immune response dominated by Th1 T cells co-producing IFNγ and IL-10, and that these cells are not associated with protection from future infection. IFNγ/IL-10 co-producing cells have been described in several parasitic infections and are hypothesized to be important in limiting CD4-mediated pathology, but they may also prevent the development of sterilizing immunity. These observations have important implications for understanding the pathophysiology of malaria in humans and for malaria vaccine development.

## Introduction

Clinical immunity to malaria eventually develops in endemic populations, but only after repeated infections with significant morbidity to both individuals and their communities [1]. Studies in regions of high malaria transmission intensity have consistently shown that the incidence of severe disease decreases considerably after the first years of life, but sterile immunity (i.e. protection against parasitemia) develops rarely if ever [2], [3]. Moreover, previously immune individuals may lose protection against symptomatic infection in the absence of continuous exposure [4], [5]. The reasons underlying the slow acquisition of clinical immunity and the failure to develop sterilizing immunity are unclear, but may include parasite diversity and evasion [6], age-related differences in immune responses [7]–[12], and/or host immunoregulatory mechanisms induced by the parasite [13]–[19]. As the incidence of malaria continues to be high in many parts of Africa despite insecticide-treated bednets and artemisinin-based combination therapy [20]–[22], there is a tremendous need to better understand mechanisms of immunity to malaria in naturally exposed populations. The identification of immunologic correlates of exposure and protection in naturally exposed children would significantly help with the rational design of vaccines and other malaria control interventions.

Both CD4^+^ and CD8^+^ T cells have been demonstrated to play an important role in protective antimalarial immunity in mouse models [23]–[30], and experimental challenge models in humans and mice strongly suggest that malaria-specific T cells contribute to protective immunity [31]–[36]. However, the identification of T cell correlates of immunity in field-based studies of naturally exposed humans has proven to be quite challenging. Prior studies employing cross-sectional or prospective cohort designs have found associations between cellular immune responses and protection from future malaria, including IFNγ responses to liver stage [37]–[40] and/or merozoite stage malaria antigens [41]–[44]. However, such studies may be confounded by the level of exposure to malaria-infected mosquitoes, which varies greatly within populations, leading subjects with lower exposure to be miscategorized as “protected” [45], [46]. Because naturally acquired immunity confers relative rather than absolute protection – manifested by a gradual decline in the incidence of clinical disease - careful quantitative outcome measures are essential, but few population-based studies of natural immunity have included careful measurement of malaria incidence over time.

Pathogen-specific T cells exhibit notable functional heterogeneity, largely dependent on the antigen and cytokine microenvironment encountered during activation, and measurement of a single parameter of T cell function (i.e. IFNγ production) may overlook others that are more critical for protection [47]. In other parasitic infections such as leishmania [48], [49] and toxoplasma [50], the functional phenotype of the CD4^+^ T cell response correlates with the success or failure to clear the pathogen. Recent observations in individuals naturally exposed to malaria suggest an important role for CD4^+^ T cell production of TNFα, with or without IFNγ, as a potential immunologic correlate of protection [51]. Conversely, CD4^+^ T cell production of the regulatory cytokine IL-10 has been implicated in modulating the severity of disease [18], [52] and may interfere with the development of protective immunity [14], [42], [53]. The role of these inflammatory and regulatory cytokines in mediating protective immunity in naturally exposed children, and in determining the balance between immunopathology and chronic repeated infection, remains unknown.

In this study we performed a detailed functional characterization of malaria-specific T cell responses among four-year-old children residing in a highly malaria-endemic region to determine whether naturally acquired T cell responses correlate with exposure to and/or protection from malaria. We hypothesized that CD4^+^ T cells producing the pro-inflammatory cytokines IFNγ and/or TNFα are associated with protection from malaria, and that T cell production of the regulatory cytokine IL-10 may interfere with the acquisition of protection. Our results suggest that the functional phenotype of the malaria-specific T cell response was heavily influenced by prior malaria exposure intensity, with CD4^+^ T cells co-producing IFNγ and IL10 dominating this response among highly exposed children. However, these IFNγ/IL-10 co-producing cells were not independently associated with protection from future malaria, and may be associated with increased risk.

## Results

### Study population and clinical outcomes

The study cohort consisted of 78 HIV-uninfected children followed from infancy through 5 years of age (Table 1). Blood for this study was drawn at four years of age (range 49–51 months), and 92% of children continued to be followed through 5 years of age. A total of 1855 incident cases of malaria were observed in this cohort through 5 years of age. All children were treated promptly with artemisinin-based combination therapy, and despite the strikingly high numbers of malaria episodes, only 4 cases of malaria were deemed “complicated” (all based on a single convulsion). No cases of severe malaria (including severe anemia) were observed. Among children with a lower prior incidence of malaria (<2 episodes per person year (ppy) between 1 and 4 years of age, n = 10), 90% lived in town; whereas among children with higher prior malaria incidence (> = 2 episodes ppy, n = 68), only 7% of children lived in town. This suggests that children with the lowest prior incidence had less exposure to malaria-infected mosquitoes. Episodes of asymptomatic parasitemia were rare in this cohort (median 1 episode per subject over the entire study period, IQR 0–4, Table 1) and the incidence of malaria declined only slightly in the year following the blood draw (from 5.7 to 5.1 episodes ppy), suggesting that effective clinical immunity had not yet emerged in most children. One child had symptomatic malaria (parasitemia with a fever requiring treatment) at the time of the blood draw, and 17 (22%) had blood smears demonstrating parasitemia.

**Table 1:** Descriptive statistics of study cohort.

Characteristic	Findings
Number of children enrolled	78
Median age in months at time of study enrollment (IQR)	5.6 (3.6–7.5)
Person-years observed from enrollment until time of blood draw	291.6
Median age in months at time of blood draw (IQR)	50.9 (48.6–51.4)
Person-years observed from blood draw until end of study	60.0
Total incident episodes of malaria	1855
Complicated malaria	4
Severe malaria	0
Median incidence of malaria	
Prior to blood draw (ppy (IQR))	5.7 (3.9–7.0)
From blood draw to end of study (ppy, IQR)	5.1 (2.5–6.9)
Median days since last episode of malaria (IQR)	37 (16–66)
Median days until next episode of malaria (IQR)	47 (20–101)
Monthly period prevalence of asymptomatic parasitemia*	
Prior to blood draw	5%
From blood draw to end of study	11%
Symptomatic malaria at the time of blood draw, n (%)	1 (1.2%)
Parasitemia at the time of the blood draw, n (%)	17 (22%)

Note: IQR, interquartile range.

Asymptomatic parasitemia defined as positive routine blood smear in the absence of fever that was not followed by the diagnosis of malaria in the subsequent seven days. Period prevalence calculated as the number of episodes/total months observed.

### The functional phenotype of malaria-specific CD4^+^ T cells is influenced by prior malaria incidence

**Figure 1:**
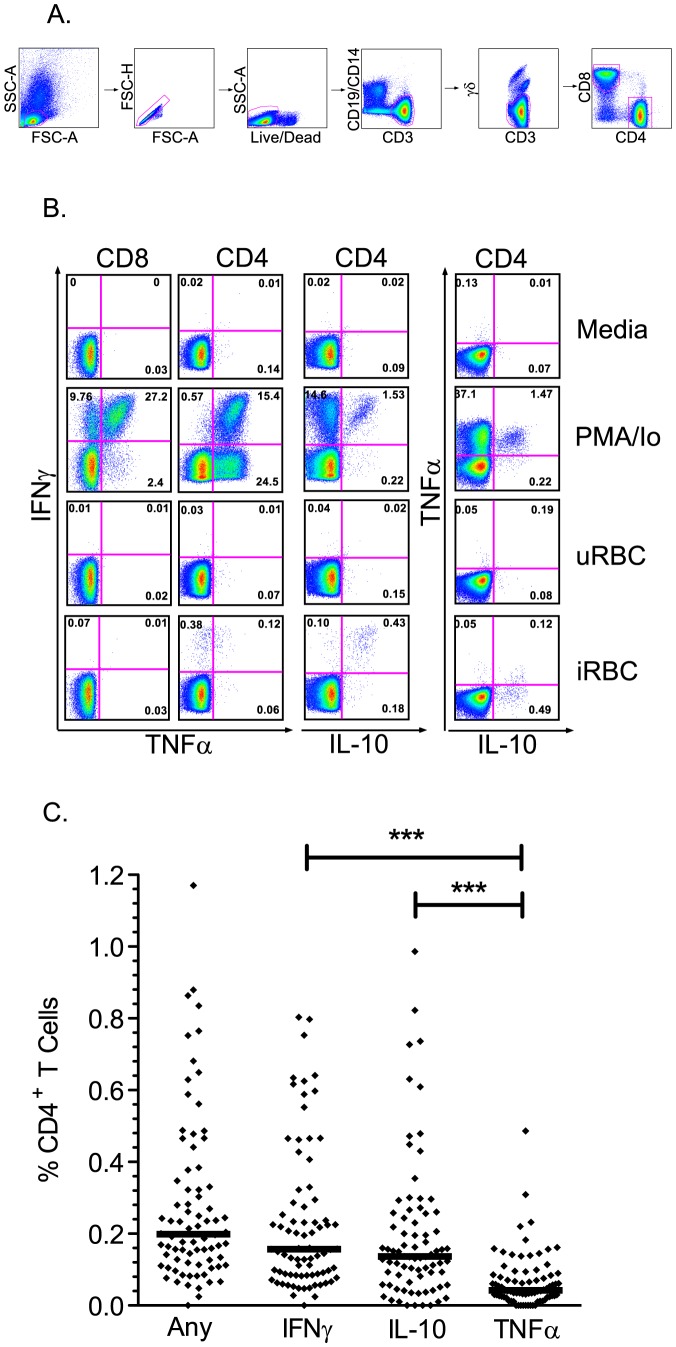
T cell responses to malaria-infected red blood cells using multiparameter flow cytometry. A. Gating strategy to identify live CD3^+^ γδ^−^ T cells. B. Intracellular cytokine assay demonstrating the T cell response of one representative malaria-exposed child to Pf-infected RBC (iRBC; bottom row), with negative controls (uRBC and media) and positive control (PMA/Io) shown in rows above. Shown are CD8 (first column) and CD4 (right 3 columns) production of IFNγ (y-axis, columns 1–3), TNFα (x-axis, columns 1–2; y-axis, column 4), and IL-10 (x-axis, column 3–4). C. The overall malaria-specific CD4^+^ T cell response (left column) is followed by the overall frequency of CD4^+^ T cells producing IFNγ, IL-10, and TNFα in all participants (n = 78, horizontal black lines indicate the median response for each group, *** *P*<0.001, Wilcoxon Rank-Sum).

To define the frequency and function of malaria-specific T cell responses, PBMC were stimulated with malaria-infected red blood cells (iRBC) and analyzed by flow cytometry for production of IFNγ, IL-10, and TNFα (Fig. 1a). The median frequency of malaria-specific CD4^+^ T cell responses producing any of these cytokines, alone or in combination, was 0.20% (IQR 0.12%–0.35%). Among all children, frequencies of CD4^+^ T cells producing IFNγ (median 0.16%) and IL-10 (median 0.14%) were significantly higher than those producing TNFα (median 0.04%, P<0.001, Fig. 1b). Production of these two cytokines largely overlapped, with a median of 83% of IL-10-producing cells also making IFNγ, and a median of 71% of IFNγ-producing cells also making IL-10. Malaria-specific production of IL-2 was tested in a subset of children (n = 44), but responses were consistently of low magnitude (median frequency 0.02%, data not shown). At the time of the assay 17 of the 78 children had positive blood smears; however there was no significant difference in the overall frequency of malaria-specific IFNγ^+^ (*P* = 0.20), TNFα^+^ (*P* = 0.29), or IL-10^+^ (*P* = 0.21) CD4^+^ T cells between children with or without parasitemia. Malaria-specific CD8 T cell responses were not observed in the peripheral blood of any of the 78 children, although this does not exclude their presence in the liver and other tissues as demonstrated by non-human primate studies [54].

**Figure 2:**
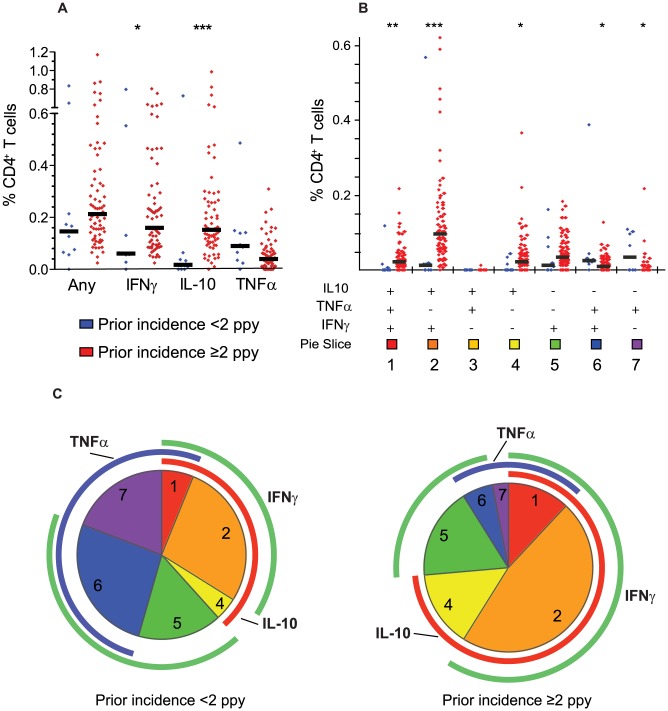
Prior malaria incidence influences function of malaria-specific CD4^+^ T cell response. A. The overall malaria-specific CD4^+^ T cell response (left column) is followed by the overall frequency of CD4^+^ T cells producing IFNγ, IL-10, and TNFα stratified by prior malaria incidence. Blue dots represent responses from children with lower prior malaria incidence (<2 episodes ppy, n = 10) and red dots represent responses from children with higher prior malaria incidence (≥2 episodes ppy, n = 68,* *P*<0.05, *** *P*<0.001, Wilcoxon Rank-Sum. Horizontal black lines indicate the median response for each group). Median frequencies of cytokine producing cells were similar in children with ≥2–5 and >5 episodes ppy (data not shown). B–C. Boolean gating of malaria-specific CD4^+^ T cells reveals 7 distinct cytokine-producing populations. Shown are the absolute frequency (B) and the relative proportion (C) of each individual combination of IFNγ, IL-10, or TNF-producing cells. Blue dots again represent responses from children with <2 prior episodes ppy, and red dots represent responses from children with ≥2 episodes ppy (* *P*<0.05, ** *P*<0.01, *** *P*<0.001, Wilcoxon Rank-Sum. Horizontal black lines indicate the median response for each group). For pie charts, blue arcs represent total proportion of CD4^+^ T cells producing TNFα; red arcs represent total proportion of CD4^+^ T cells producing IL-10; and green arcs represent total proportion of CD4^+^ T cells producing IFNγ. The proportion of IFNγ^−^/IL-10^+^/TNFα^−^ (population 3) producing cells is <0.01% of the total malaria-specific response, and thus does not have a visible corresponding pie slice.

The pattern of cytokine production by malaria-specific CD4^+^ T cells was noted to differ markedly based on children's prior incidence of malaria (Fig. 2a–c). Both IL-10-producing CD4^+^ T cells and IFNγ-producing CD4^+^ T cells were present at higher frequencies among children with a higher prior incidence of malaria (≥2 episodes ppy) than among those with a lower prior incidence (<2 episodes ppy, *P*<0.001 and *P* = 0.02, respectively, Fig. 2a). Most strikingly, CD4^+^ T cells co-producing IFNγ and IL-10 dominated the response among children with higher prior incidence, but were virtually absent among lower incidence children (*P*<0.001, Fig. 2b). Production of TNFα followed the opposite pattern, with higher frequencies of TNFα^+^/IL10^−^ CD4^+^ T cells observed among children with lower prior incidence than among those with a higher prior incidence (*P* = 0.003, Fig. 2b). Interestingly, despite these differences in cytokine production profiles, the overall frequency of malaria-specific CD4^+^ T cells (i.e. those producing any cytokine) did not statistically differ between the higher and lower incidence groups (*P = *0.13).

We also analyzed the relationship of prior malaria incidence with the “composition” of the malaria-specific response (i.e. the proportion of each cytokine combination amongst the total malaria-specific CD4^+^ T cell population), and found similar results. Among children with <2 episodes ppy, TNFα-producing CD4^+^ T cells (including TNFα single-producers and IFNγ/TNFα double producers) comprised a greater proportion of the malaria-specific response than among children with ≥2 prior episodes ppy, whereas in children with a higher prior malaria incidence, IL-10-producing CD4^+^ T cells (including IL-10 single-producers and IFNγ/IL-10 double producers) comprised a far greater fraction of the malaria-specific response (*P*<0.001, Fig. 2c). There was no significant difference in the proportion of IFNγ-producing CD4^+^ T cells between children with higher and lower incidence. These findings suggest that the functional phenotype of the malaria-specific CD4^+^ T cell response differs according to prior exposure, and that with more prior episodes, the overall response is more regulatory (IL-10 producing) and less inflammatory (TNFα producing).

### IFNγ/IL-10 co-producing CD4^+^ T cells correlate with recent malaria exposure

**Figure 3:**
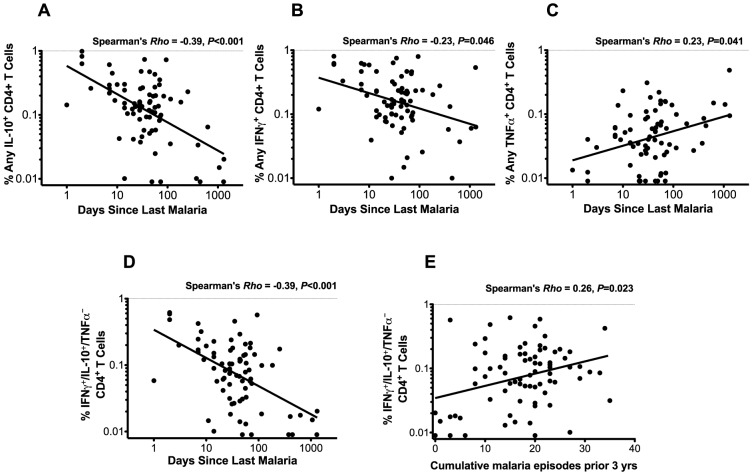
CD4^+^ T cell functions and relationship with recent and cumulative malaria infection. The frequencies of CD4^+^ T cells producing any IL-10 (A) and any IFNγ (B) are inversely associated with days since last malaria episode (Spearman's *Rho* = −0.39, *P*<0.001; *Rho* = −0.23, *P* = 0.046, respectively). Frequencies of CD4^+^ T cells producing any TNFα (C) are positively correlated with days since last malaria episode infection (Spearman's *Rho* = 0.23, *P* = 0.041). Frequencies of IFNγ^+^/IL-10^+^/TNFα^−^ CD4^+^ T cells are inversely associated with days since last malaria episode (D, Spearman's *Rho* = −0.39, *P*<0.001) and positively associated with the cumulative number of episodes in the prior 3 years (E, Spearman's *Rho* = 0.26, *P* = 0.023).

While the data above demonstrate that there is a strong relationship between the functional phenotype of malaria-specific CD4^+^ T cells and prior malaria history, we wished to determine whether this phenotype was influenced by the time elapsed since the most recent malaria episode, the cumulative number of prior malaria episodes, or both, as these parameters are both logically and statistically related (Spearman's *Rho* = −0.46, *P*<0.001). We observed a strong inverse correlation between the frequency of IFNγ^+^/IL-10^+^/TNFα^−^ CD4^+^ T cells and the duration since the last episode of malaria (Spearman's *Rho* = −0.39, *P*<0.001, Fig. 3d), with more recent malaria associated with a higher frequency of these co-producing cells, as well as a positive correlation with the total cumulative number of prior episodes (Spearman's *Rho* = 0.23, *P* = 0.04, Fig. 3e). However, when assessed in a multivariate model, the frequency of malaria-specific IFNγ/IL-10 co-producing CD4^+^ T cells remained strongly associated with the duration since malaria, whereas the total prior incidence was no longer significant. Similar results were observed for total IL-10 (Fig. 3a) and total IFNγ-producing (Fig. 3b) populations, and when assessing the duration since last episode of parasitemia (data not shown). Interestingly, the opposite relationship was observed between total TNFα^+^ producing cells and the duration since last episode of malaria, with more recent malaria associated with a lower frequency of TNFα -producing cells (Spearman's *Rho* = 0.23, *P* = 0.041, Fig. 3c). Further, there was no significant correlation between the number of cumulative prior malaria episodes and TNFα^+^ producing cells. Together these data suggest that recency of malaria infection, rather than the total number of past episodes, exerts a dominant influence on the functional phenotype of malaria-specific CD4^+^ T cells. Similar findings were obtained when analyzing the “composition” (i.e. the proportion of responding cells producing IFNγ, TNFα, and/or IL10) of the malaria-specific response and duration since last malaria infection.

### Malaria-specific CD4^+^ T cells are not independently associated with protection from malaria

Protection from clinical malaria in naturally exposed individuals can be defined using a number of outcomes, including a delayed time to reinfection [37], [38], [41]–[43], [51], a decreased incidence of malaria over time [53], and/or a decreased probability of clinical disease once parasitemic [46]. In all cases, identification of immune correlates of protection is challenging due to the difficulty of distinguishing protection from a lack of exposure to malaria-infected mosquitos [45], [46]. To address this, we assessed the relationship between malaria-specific T cell functional subsets and protection from malaria, while adjusting for prior malaria (duration since last episode and/or cumulative number of prior episodes) as a surrogate measure of exposure intensity. We also evaluated potential associations with the overall prevalence of asymptomatic parasitemia, as clinical immunity to malaria is normally characterized by a transition from symptomatic to asymptomatic disease [3].

In univariate Cox proportional hazards analysis evaluating time to next episode of malaria, a higher frequency of CD4^+^ T cells producing any IFNγ or IL10, or the combinations IFNγ^+^/IL-10^+^/TNFα^−^ and IFNγ^−^/IL-10^+^/TNFα^−^ was associated with a significantly increased hazard of malaria (Table 2, left columns). However following adjustment for surrogates of exposure intensity (duration since last episode of malaria and/or cumulative prior malaria episodes) in a multivariate model, none of these associations remained significant. Similar relationships were observed when we analyzed the total malaria incidence in the year following the assay in a multivariate regression model (Table 2, middle columns). However, in this analysis both IFNγ^+^/IL-10^+^/TNFα^−^ (IRR 1.40 per 10 fold increase, *P = *0.038) and any IL-10-producing CD4^+^ T cells (IRR 1.41 per 10 fold increase, *P = *0.039) remained independently associated with an increased risk of malaria after controlling for duration since last malaria infection. Nearly identical results were obtained when analyzing the total composition of cytokine producing cells: both the fraction of IFNγ^+^/IL-10^+^/TNFα^−^ and any IL10^+^ cells among all cytokine-producing cells were associated with increased malaria risk (IRR 1.47, P = 0.038 and 1.40, P = 0.039 per 50% increase in fraction of responding cells, respectively). Together, these data suggest that the dominant population of malaria-specific CD4^+^ cells, which co-produce IFNγ and IL-10, are not associated with protection from future malaria, and may in fact be associated with an increased risk of malaria.

**Table 2:** Magnitude of malaria-specific CD4^+^ T cell responses and protection from symptomatic malaria.

	Time until malaria				Future incidence of malaria				Prevalence of asymptomatic parasitemia			
	Univariate		Multivariate*		Univariate		Multivariate*		Univariate		Multivariate*	
% CD4^+^ T cells (Log_10_)	HR	*P*	HR	*P*	IRR	*P*	IRR	*P*	PRR	*P*	PRR	*P*
**1)** IFNγ^+^/IL-10^+^/TNFα^+^	1.79	0.051	1.14	0.671	1.42	0.085	1.29	0.134	0.51	0.111	0.43	0.037
**2)** IFNγ^+^/IL-10^+^/TNFα^−^	2.22	0.001	1.73	0.083	1.65	0.002	**1.40**	**0.038**	0.86	0.703	0.74	0.482
**3)** IFNγ^−^/IL-10^+^/TNFα^+^	n/a	n/a	n/a	n/a	n/a	n/a	n/a	n/a	n/a	n/a	n/a	n/a
**4)** IFNγ^−^/IL-10^+^/TNFα^−^	1.73	0.047	1.53	0.198	1.43	0.059	1.24	0.216	1.63	0.247	1.94	0.130
**5)** IFNγ^+^/IL-10^−^/TNFα^−^	1.32	0.365	1.24	0.532	1.02	0.917	1.04	0.815	1.23	0.649	1.30	0.567
**6)** IFNγ^+^/IL-10^−^/TNFα^+^	0.65	0.249	1.52	0.286	0.89	0.649	1.32	0.199	0.39	0.052	0.48	0.152
**7)** IFNγ^−^/IL-10^−^/TNFα^+^	0.31	0.015	1.25	0.725	0.44	0.004	0.73	0.297	0.43	0.070	0.61	0.422
Any IFNγ^+^	1.84	0.039	1.74	0.091	1.44	0.076	1.41	0.062	0.60	0.28	.60	0.283
Any IL-10^+^	2.26	0.001	1.64	0.117	1.70	0.001	**1.41**	**0.039**	1.00	0.99	.88	.787
Any TNFα^+^	0.90	0.694	1.49	0.161	0.94	0.720	1.20	0.254	1.20	0.254	**0.38**	**0.004**

Note: HR: Hazard Ratio; IRR: incidence rate ratio; PRR: prevalence rate ratio. Numbered rows refer to cell populations described in Figure 2. Associations in row 3 are not applicable because these responses were undetectable.

Multivariate models controlled for duration since last malaria infection. Similar results were obtained when controlling for cumulative episodes over prior 3 years and for the presence or absence of parasitemia.

We next assessed the relationship of TNFα^−^ producing CD4^+^ T cells with protection. In RTS/S vaccine recipients, malaria-specific CD4^+^ T cells producing TNFα in the absence of IFNγ or IL-2 have recently been shown to correlate with protection from malaria infection [55]. In our cohort, a greater frequency of malaria-specific CD4^+^ T cells producing TNFα alone (IFNγ^−^/IL-10^−^/TNFα^+^) was associated with a significantly reduced hazard of developing malaria (HR 0.31, *P* = 0.015 per 10 fold increase) and lower prospective incidence (IRR 0.44, *P* = 0.004 per 10 fold increase) in univariate analysis, but in multivariate models controlling for duration since malaria and/or cumulative prior malaria episodes, these associations were no longer significant (Table 2). Interestingly, however, the frequency of malaria-specific CD4^+^ T cells producing any TNFα was inversely associated with the monthly prevalence of asymptomatic parasitemia, even after controlling for duration since last episode of malaria and/or cumulative prior malaria episodes (PRR 0.41 per 10 fold increase, *P* = 0.011). Thus, the absence of malaria-specific CD4^+^ T cells producing TNFα may be associated with the phenotype of asymptomatic infection.

### IFNγ/IL-10 co-producing cells express *T-bet* and are of an effector memory phenotype

**Figure 4:**
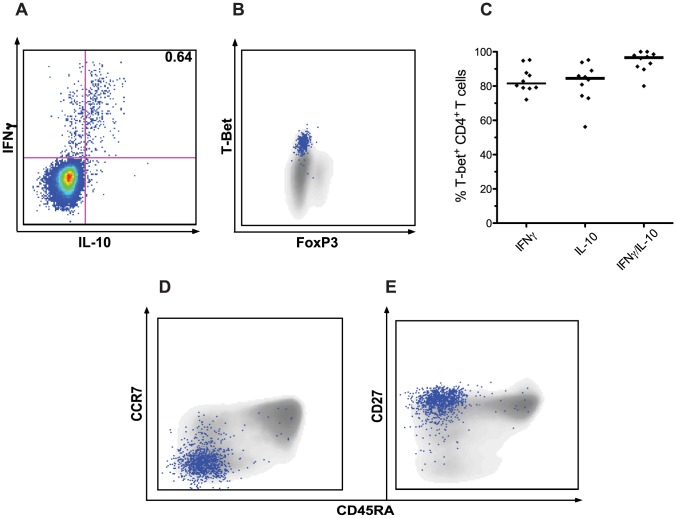
CD4^+^ T cells co-producing IFNγ and IL-10 express *T-bet* and are of an early effector memory phenotype. CD4^+^ T cells were analyzed for transcription factor expression and maturational phenotype. Panel 4A shows the proportion of CD4^+^ T cells co-producing IFNγ/IL-10 in response to iRBC stimulation for one representative child (upper right quadrant)) and panel 4B shows intranuclear transcription factor staining with T-bet and FoxP3 of all CD4^+^ T cells (grey) with IFNγ/IL-10 CD4^+^ T cells overlayed (4B, blue dots). Panel 4C shows the percentage of iRBC-stimulated IFNγ^+^, IL-10^+^, and IFNγ/IL-10 co-producing CD4^+^ T cells staining for intranuclear T-bet (n = 10). CD4^+^ T cells were also analyzed for cell surface maturation markers CD45RA (x axis, panels 4D–E), CCR7 (4D), and CD27 (4E). The total CD4^+^ T cell population is shown in grey, with IFNγ/IL-10 co-producing CD4^+^ T cells overlayed as blue dots.

Although IL10 production by T cells was initially believed to occur predominantly within Th2 and FoxP3^+^ T_reg_ CD4^+^ T cell subsets, it is now known that additional subsets, including cells expressing the Th1 master regulator *T-bet*, produce IL-10 under conditions of continuous antigen exposure [56], [57]. We assessed transcription factor expression within the dominant population of malaria-specific IFNγ/IL-10 co-producing cells (Fig. 4a) and found that these cells uniformly were TBet^+^ and FoxP3^−^ (Fig. 4b–c). These IFNγ/IL-10 co-producing CD4^+^ T cells were predominantly of an early effector memory phenotype (CD45RA−, CCR7− CD27+; Fig. 4d–e).

CD4^+^ T cell IFNγ/IL-10 responses to the polyclonal mitogen PMA/Io have previously been shown to correlate with relative protection against severe malaria [52]. We therefore compared the response to iRBC and PMA/Io stimulation, and found a strong correlation between the frequency of IFNγ/IL-10 double producing CD4^+^ T cells following iRBC or PMA stimulation (Spearman's *Rho* = 0.88, *P*<0.001, Supplemental Fig. S1). As PMA/Io stimulation is thought to induce cytokine production by recently activated cells, these data suggest that this mitogen stimulates cytokine production by malaria-specific T cells that have recently seen their cognate antigen.

### Plasma IL-10 levels are elevated during malaria infection but do not correlate with the frequency of IL-10 producing CD4^+^ T cells

IL-10 levels measured concurrently in plasma were significantly higher among children with parasitemia at the time of the blood draw compared with children with no parasitemia (median 30.4 pg/ml vs 11.4 pg/ml, *P* = 0.0035), consistent with prior reports [58]–[61]. Similar to IL-10 producing CD4^+^ T cells, plasma IL-10 strongly correlated with recent malaria (Spearman's *Rho* = 0.30, *P* = 0.009, Supplemental Fig. S2a). However plasma IL-10 levels did not correlate with the frequency of total IL-10 producing CD4^+^ T cells (Spearman's *Rho*  = 0.11, *P* = 0.35, Supplemental Fig. S2b), suggesting that additional cell types, including cells of the myeloid lineage, may contribute to plasma IL-10 levels during malaria infection [19].

### Impaired malaria-specific CD4^+^ T cell proliferation in heavily exposed children is partially reversed by IL-10 blockade

**Figure 5:**
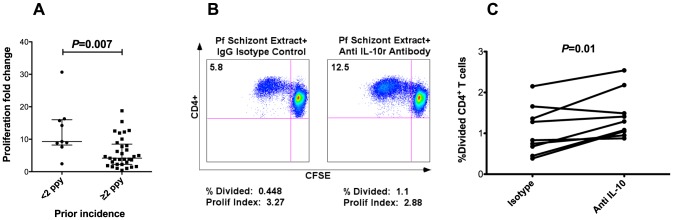
CD4^+^ T cell proliferation impaired in setting of heavy prior exposure. A. The proliferation fold change (fraction of CFSE-lo cells following PfSE stimulation vs uRBC stimulation) is significantly reduced in children with higher prior malaria exposure (≥2 episodes ppy, n = 33) vs children with low malaria exposure (<2 episodes ppy, n = 9, *P* = 0.007, Wilcoxon Rank Sum. Horizontal lines show medians for each group with 95% CI). B. Impact of IL-10 blockade on CD4^+^ T cell proliferation following PfSE stimulation in one representative subject. The left panel shows CFSE dilution following PfSE stimulation with addition of isotype control, and the right panel shows CFSE dilution following PfSE stimulation with addition of anti IL-10 receptor α blocking antibody. C. Change in the percent of CD4^+^ T cells divided following isotype control vs anti IL-10 receptor α blocking antibody in a subset of 9 children from whom additional cells were available (fold change 1.7, *P* = 0.01).

Immunomodulation through downregulation of antigen-specific CD4^+^ T cell proliferative responses has been described in the context of several chronic parasitic infections [62]–[65], as well as chronic viral infections that result in persistent antigenemia [66], [67]. We assessed proliferation of malaria-specific CD4^+^ T cells by measuring CFSE dilution following stimulation with schizont extract (PfSE) in a subset of children (n = 42). A significant inverse correlation was observed between malaria-specific CD4^+^ T cell proliferation and cumulative prior incidence (Spearman's Rho = −0.39, *P* = 0.011; Fig. 5a), suggesting that heavy antigen exposure may result in a proliferative defect in malaria-specific CD4^+^ T cells. We also observed an inverse correlation between CD4^+^ T cell proliferation following PfSE stimulation and the frequency of IFNγ/IL-10 co-producing CD4^+^ T cells (Spearman's Rho = −0.31, *P* = 0.049). It has previously been suggested that IFNγ/IL-10 co-producing CD4^+^ T cells may play an autoregulatory role through suppression of proliferative responses in an IL-10 mediated manner [68]. We therefore assessed whether *in vitro* IL10 blockade would reverse the observed proliferative defect. The ability of CD4^+^ T cells to proliferate in response to PfSE was partially restored in 8 of 9 subjects upon blockade of IL-10 receptor alpha (fold change 1.7, *P* = 0.01, Fig. 5b–c), suggesting that the CD4^+^ T cell proliferative defect observed in heavily exposed children may be in part due to IL-10 mediated suppression.

## Discussion

In this cohort of young children living in an area of very high transmission intensity in Uganda, very little evidence of clinical immunity had emerged by five years of age. In this setting, the functional phenotype of the malaria-specific CD4^+^ T cell response was significantly influenced by prior malaria exposure; with less prior malaria, the overall malaria-specific CD4^+^ T cell response was more inflammatory (TNFα-producing), but with heavier exposure, the overall malaria-specific response was more regulatory (IL-10 producing). To our knowledge, this is the first study to show that Th1 IFNγ/IL-10 co-producing cells constitute the dominant population of CD4^+^ T cells responding to malaria in heavily exposed children. Moreover, we found no evidence that these IFNγ/IL-10 co-producing cells were associated with protection from future malaria.

Interest in IFNγ/IL-10 co-producing Th1 cells has increased in recent years as these cells have been found to be important regulators of the immune response to several infectious, allergic, and autoimmune diseases [18], [49], [50], [52], [56], [69], [70]. In a murine model of *Toxoplasma gondii*, IFNγ produced by these cells was shown to be required for pathogen eradication, and concomitant production of IL-10 was vital for the resolution of the inflammatory response and to prevent tissue pathology [50]. However, in a murine model of *Leishmania major*, co-production of IL-10 by Th1 cells prevented pathogen eradication, contributing to chronic infection [49]. These data suggest that IL-10 co-production by Th1 T cells may help prevent immunopathology, but this may come at the cost of chronic pathogen persistence [71].

IL-10 levels are increased during malaria infection [58], [59], [61] and this regulatory cytokine is thought to play a key role in dampening proinflammatory responses and preventing the development of severe malarial anemia and cerebral malaria [72]. In mice, Th1 cells were elegantly shown to be the major producer of IL-10 and were critical for limiting the pathology associated with malaria infection [18]. T cell production of IL-10 has also been described in reports of human malaria infection [14], [52], [73]–[77]. Plebanski and colleagues described a switch in production from IFNγ to IL-10 in CD4^+^ T cells from Gambian adults stimulated with altered peptide ligands of the circumsporozoite protein, with an associated suppression of proliferative responses in vitro [14]. T cells co-producing IFNγ/IL-10 following nonspecific PMA/ionomycin stimulation were described in the context of acute malaria infection [73], and were also more abundant among children with uncomplicated rather than severe malaria [52], consistent with a role in modulating inflammation. More recently, Gitau and colleagues described malaria-specific co-production of IFNγ and IL-10 following stimulation of CD4^+^ T cells with a variety of expressed PfEMP variants, although these co-producing cells represented a minor fraction of the total antigen-specific CD4^+^ T cell response [75]. The potential role that malaria-specific IFNγ/IL-10 co-producing CD4^+^ T cell cells play in mediating or inhibiting protective immunity in humans has not thus far been investigated [77].

We observed that CD4^+^ T cells co-producing IFNγ/IL-10 dominate the T cell response to malaria in heavily exposed children, and that the overall frequency and proportion of these cells among malaria-specific T cells was strongly correlated with recent exposure to malaria, more so than cumulative prior exposure. These IFNγ/IL-10 co-producing cells express *T-bet*, indicating that they have differentiated along the Th1 pathway. The dominance of this functional phenotype among malaria-specific T cells has not previously been reported, and may be related to the unusually high malaria exposure intensity of our cohort, as this cell population was of much lower frequency among children with <2 malaria episodes per year. Further, frequencies of IL-10–producing and IFNγ/IL10 co-producing cells were not associated with protection from future malaria after controlling for recent and/or cumulative prior malaria, but were instead associated with an increased risk of cumulative malaria in the year following the assay, although this may be due to the inability to fully adjust for the level of environmental exposure to malaria using clinical surrogates such as prior malaria incidence.

We further observed that heavy malaria exposure was associated with a decreased ability of CD4^+^ T cells to proliferate in response to malaria antigens, and that this impaired proliferation is partially reversed by IL-10 blockade. These data are consistent with *in vitro* studies of recently activated IL7R−, CD25−, CD4^+^ T cells which co-produce IFNγ and IL-10 and limit CD4^+^ T cell proliferation through IL-10 dependent mechanisms [68]. In addition, prior studies have shown that IL-10 blockade increases malaria-specific IFNγ cytokine production in filaria-coinfected individuals [78] and in cord blood mononuclear cells from neonates born to mothers exposed to malaria [79]. A similar IL10-dependent functional impairment of CD4^+^ T cells has been described in other infections such as HIV that are characterized by chronic high-level antigen stimulation [80], [81].

Together, these data are consistent with the hypothesis that IFNγ/IL-10 co-producing CD4^+^ T cells primarily function to limit the immunopathology associated with malaria infection – including cerebral malaria, anemia, and death - through autoregulation of CD4^+^ T cell proliferation and cytokine production. A similar role has been attributed to IL-10-producing Th1 cells in other parasitic diseases characterized by heavy continuous antigen exposure [49], [50], with evidence that IL-10 produced by Th1 effector cells acts through a negative feedback loop to regulate CD4^+^ T cell responsiveness, limiting inflammation and tissue pathology at the cost of impaired pathogen clearance [56], [71]. It is possible that unmeasured confounders, such as helminthic co-infections, may have been unequally represented in the high and low-incidence groups, particularly as the lower incidence children were more likely to reside in town. However routine deworming was performed in all study subjects every 3–6 months, lessening the likelihood that co-infection with helminths explains our findings. Further studies are needed to determine if IL-10-producing Th1 cells contribute to pathogen persistence, and to the failure of humans to develop sterile protective immunity to malaria.

In addition, we found that children with the fewest prior episodes of malaria were significantly more likely to have malaria-specific production of TNFα without IL-10, and that the absence of this inflammatory cytokine was associated with the phenotype of asymptomatic infection. Studies in murine models have shown that TNFα plays an important role in inhibiting the development of hepatic stages of malaria [82], [83]. Importantly, a recent study of RTS/S vaccine recipients identified antigen-specific CD4^+^ T cell production of TNFα as a correlate of protection in vaccinees [55]. In contrast to that study, we found no evidence of protection after controlling for prior malaria, though we did observe that asymptomatic infection was inversely associated with the frequency of TNFα producing CD4^+^ T cells, independent of prior malaria. Together our data suggest that production of this inflammatory cytokine may decrease with increasing cumulative malaria exposure, enabling a transition to asymptomatic infections.

A notable strength of this study was the availability of comprehensive malaria clinical histories spanning from early infancy to the time of the immunologic assessment, plus one additional year thereafter, which enabled us to assess for T cell correlates of both exposure to and protection from malaria. Several prior studies have reported correlations between T cell responses or IL-10 production and protection from malaria in naturally exposed children [37], [42], [53], but such studies have generally been unable to adequately account for prior malaria exposure. While we did observe associations, both positive and negative, between malaria-specific CD4^+^ T cells of varying functional phenotypes and the risk of future malaria, most of these associations were not significant after adjusting for recent or cumulative prior episodes of malaria, surrogates for the level of ongoing exposure to malaria-infected mosquitos. Hence the failure to account for malaria exposure intensity may lead to spurious associations with protection. Although we did not identify T cell phenotypes that were associated with protection from future malaria, this may be related to the young age of children in this cohort, as there was little evidence that clinical immunity had developed prior to 5 years of age. Future longitudinal studies examining responses in older children and adults, incorporating more precise entomological measurements of malaria exposure, are underway.

In conclusion, among naturally exposed children living in a high endemicity setting, malaria-specific CD4^+^ T cells were present in the vast majority of children, and their functional phenotype differed greatly based on the level of prior exposure to malaria, in particular the duration of time since last infection. IFNγ/IL-10 co-producing Th1 cells dominated the CD4^+^ T cell response to malaria in these heavily exposed children, but were not associated with protection from future infection. These CD4^+^ T cells may play important immunomodulatory roles in the pathogenesis of malaria in childhood.

## Methods

### Study site, participants, and follow-up procedures

Samples for this study were obtained from children enrolled in the Tororo Child Cohort (TCC) in Tororo, Uganda, a rural district in south-eastern Uganda with an entomological inoculation rate (EIR) estimated at 379 infective bites per person year (PPY) in 2012 [20]. Details of this cohort have been described elsewhere, and the sub-study described in this report includes only HIV-uninfected children born to HIV-uninfected mothers [20], [84]–[87]. Briefly, children in the TCC were enrolled at infancy (median 5.5 months of age) and followed for all medical problems at a dedicated study clinic open seven days a week. Monthly assessments were done to ensure compliance with study protocols and perform routine blood smears. All children were prophylactically dewormed with mebendazole every 3–6 months per Ugandan Ministry of Health guidelines [88]. Children who presented with a documented fever (tympanic temperature ≥38.0°C) or history of fever in the previous 24 hours had blood obtained by finger prick for a thick smear. If the thick smear was positive for malaria parasites, the patient was diagnosed with malaria regardless of parasite density, and given artemisinin-based combination therapy for treatment of uncomplicated malaria. Children were followed until 5 years of age unless prematurely withdrawn.

Incident episodes of malaria were defined as all febrile episodes accompanied by any parasitemia requiring treatment, but not preceded by another treatment in the prior 14 days [20]. The incidence of malaria was calculated as the number of episodes per person years (ppy) at risk. Asymptomatic parasitemia was defined as a positive routine blood smear in the absence of fever that was not followed by the diagnosis of malaria in the subsequent seven days, and was reported as a count outcome as it was measured via monthly surveillance. The period prevalence of asymptomatic parasitemia was calculated as the number of episodes/total months observed.

### Ethical approval

Written informed consent was obtained from the parent or guardian of all study participants. The study protocol was approved by the Uganda National Council of Science and Technology and the institutional review boards of the University of California, San Francisco, Makerere University and the Centers for Disease Control and Prevention.

### Sample collection and processing

At approximately 4 years of age, ∼6–10 mls of whole blood was obtained from each subject in acid citrate dextrose tubes. Plasma was collected, and peripheral blood mononuclear cells (PBMC) were isolated by density gradient centrifugation (Ficoll-Histopaque; GE Life Sciences). PBMC were cryopreserved in liquid nitrogen and shipped to our laboratory in San Francisco for additional studies.

### Malaria antigens

*Plasmodium falciparum* blood-stage *3D7* parasites were grown by standard methods and harvested at 5–10% parasitemia. Red blood cells infected with mature asexual stages were purified magnetically, washed, and cryopreserved in glycerolyte prior to use (iRBC). Uninfected RBCs (uRBC) were used as controls. To assess the impact of parasite diversity on T cell responses, responses to iRBCs prepared from 4 distinct Tororo field strains were compared to iRBC prepared from 3D7. Responses to the 4 field strains were very similar, indicating that parasite diversity does not significantly influence the T cell response magnitude (Supplemental Fig. S3). Schizont extracts (PfSE) for use in proliferation assays [89] were prepared by 3 freeze-thaw cycles of iRBC in liquid N_2_ for freezing and 37°C water bath for thawing, then resuspended in R10 media and stored at −20°C until use.

### Intracellular cytokine staining

Thawed PBMC were rested overnight in 10% fetal bovine serum (Gibco) and counted prior to stimulation with uRBC, iRBC, media, or phorbol miristate acetate/calcium ionophore (PMA/Io) at 1×10^6^ cells/condition. An E:T ratio of 1∶3 was used with uRBC and iRBC [90]. Anti-CD28 and –CD49d were added for costimulation (0.5 µg/ml, BD Pharmingen). Brefeldin-A and Monensin (BD Pharmingen) were added at 6 hours of incubation at a final concentration of 10 µg/ml to inhibit cytokine secretion. At 24 hours of incubation, cells were washed, fixed and permeabilized per standard protocols (Invitrogen/Caltag; Ebioscience fix/perm reagents used for nuclear transcription factor analysis).

Surface and/or intracellular staining of PBMC was done with standard protocols [91], [92] using the following antibodies for the primary analysis: Brilliant violet 650-conjugated CD4 (Biolegend), PerCP–conjugated anti-CD3, APC-H7-conjugated CD8, PE-Cy7-conjugated IFNγ, PE-conjugated anti-IL-10, and FITC-conjugated TNFα (BD Pharmingen). Alexa 700-conjugated CD14 and CD19, APC-conjugated anti-γδ (Biolegend), and Live/dead aqua amine (Invitrogen) were included as exclusion gates to reduce unwanted nonspecific antibody binding when measuring antigen-specific T cell populations [93]. Additional experiments utilized Brilliant violet 421-conjugated anti-IL-2, Brilliant violet 605-conjugated CD45RA, Brilliant violet 710-conjugated CD27 (Biolegend), APC-conjugated CCR7 (R&D Systems); eFluor 660-conjugated T-bet and FITC-conjugated FoxP3 (Ebioscience).

### CFSE proliferation assay

Thawed PBMC were rested for one hour, washed in 10% Human AB media (Gemini), and 3–6×10^6^ PBMC were labeled with 1 ml of 1.25 µM 5,6-carboxyfluorescein diacetate succinimidyl ester (CFSE; Molecular Probes) for seven minutes. CFSE-labeled PBMC were incubated in 96-well, deep-well culture plates (Nunc, Roskilde, Denmark) at a density of 10^6^ PBMC per well at a final volume of 1 ml for 7 days. In a subset of patients, CFSE-labeled PBMC were incubated with antigen in the presence of IL-10 receptor alpha chain (IL-10Rα) blocking antibody (clone 37607; R&D Systems) or IgG1 isotype control antibody at 10 µg/mL. Antigens tested included media, phytohemagglutinin (PHA; 5 µg/mL; Sigma-Aldrich), uRBC, or PfSE at an E:T ratio of 1∶3 schizont equivalents. At day 7 cells were treated with 100 units DNase I (Invitrogen) in culture medium at 37°C for 10 min, washed, and stained with surface antibodies (PerCP–conjugated anti-CD3, APC-H7-conjugated CD8 (BD Pharmingen), Brilliant violet 650-conjugated CD4, Alexa 700-conjugated CD14 and CD19, and APC-conjugated anti-γδ (Biolegend)) before acquisition.

### Flow cytometry data analysis

Flow cytometry profiles were gated on CD3^+^, γδ-negative lymphocytes, and 200,000 to 300,000 events were collected. Samples were analyzed on an LSR2 three laser flow cytometer (Becton Dickinson) with FACSDiva software. Color compensations were performed for each patient's PBMC using beads or samples single stained for each of the fluorochromes used. Data were analyzed using FlowJo (Tree Star, San Carlos, CA) and Pestle (version 1.7)/SPICE (version 5.3; M. Roederer, Vaccine Research Center, National Institute of Allergy and Infectious Diseases, National Institutes of Health, Bethesda, MD). In experiments with CFSE-labeled cells, the ratio of CFSE-lo cells following PfSE stimulation vs uRBC stimulation was calculated and reported as the proliferation fold change. The FlowJo Proliferation Platform provided additional information about the division characteristics of CD4^+^ T cells. To examine the effect of IL-10 blockade on proliferation, these parameters for CD4^+^ T cells were generated in samples that had been stimulated with PfSE plus anti-IL-10Rα and compared to the values obtained from samples stimulated with PfSE plus isotype control.

### Plasma IL-10

Plasma levels of IL-10 were measured by dual Ab sandwich-ELISA kits, according to manufacturer's instruction (R&D Systems, Minneapolis, MN). Each sample was tested in duplicate, and cytokine concentrations were calculated using a standard curve generated from recombinant cytokines. Cytokine values were expressed as picograms (pg) per milliliter.

### Statistical methods

All statistical analyses were performed using Prism 4.0 (GraphPad), STATA version 12 (College Station), or SPICE v.5.3 (NIAID). Frequencies of malaria-specific cytokine producing T cells (alone or in combination) are reported after background subtraction of the frequency of the identically gated population of cells from the same sample stimulated with control. Background-subtracted responses were consider positive if >0.01% parent population [94]. Comparisons of cytokine frequencies between prior malaria incidence groups were done using the Wilcoxon rank sum test, and the Wilcoxon signed-rank test was used to compare paired data. Statistical analyses of global cytokine profiles (pie charts) were performed by partial permutation tests using the SPICE software [94] . Continuous variables were compared using Spearman correlation. For multivariate regression models, non-normal variables were log-transformed. To allow for nonlinear relationships between clinical exposure variables and immunologic outcomes, we fit linear splines with knots chosen to best represent observed relationships. Associations between immune parameters and time to next malaria episode were evaluated using the Kaplan-Meier product limit formula, and a multivariate cox proportional hazards model was used to adjust for surrogates of malaria exposure found to be associated with these parameters (duration since last episode of malaria and/or cumulative episodes in the prior 3 years). Negative binomial regression was used to estimate associations between immune parameters and the prospective incidence of malaria in the following year (incidence rate ratios, IRR) and prevalence of asymptomatic parasitemia during the entire study period (prevalence rate ratios, PRR), adjusting for prior malaria as above. Two-sided p-values were calculated for all test statistics and *P*<0.05 was considered significant.

## Supporting Information

Figure S1 **Relationship between iRBC and PMA/Io stimulation.** The frequency of iRBC-stimulated CD4^+^ T cells producing IFNγ/IL-10 is strongly correlated with the frequency of PMA/Io-stimulated CD4+ T cells co-producing these cytokines (Spearman's *Rho* = 0.86, *P*<0.0001).
(EPS)

Figure S2 **Relationship of Plasma IL-10 levels with malaria and CD4^+^ T cells.** Plasma IL-10 is inversely associated with days since last episode of malaria (A, Spearman's *Rho* = −0.30, *P* = 0.009). There is no significant association between plasma IL-10 levels and the frequency of IL-10 producing CD4+ T cells (B, Spearman's *Rho* = 0.11, *P* = 0.35).
(EPS)

Figure S3 **T cell responses to malaria-infected red blood cells comparing responses to 3D7 and field isolates.** Intracellular cytokine staining assay demonstrating the CD4^+^ T cell response of a malaria-exposed child to several strains of field isolates, with negative control (uRBC), positive control (PMA/Io), lab-adapted 3D7, and four distinct field isolates from Tororo, Uganda (provided courtesy of Dr. Philip Rosenthal). Plots are gated on CD4^+^ T cells and shown are frequencies of CD4^+^ T cells making IFNγ alone (top left quadrant), IFNγ and IL-10 (top right quadrant), and IL-10 alone (bottom right quadrant).
(EPS)
